# Usability and Acceptability of a Smartphone App to Assess Partner Communication, Closeness, Mood, and Relationship Satisfaction: Mixed Methods Study

**DOI:** 10.2196/14161

**Published:** 2020-07-06

**Authors:** Shelby L Langer, Neeta Ghosh, Michael Todd, Ashley K Randall, Joan M Romano, Jonathan B Bricker, Niall Bolger, John W Burns, Rachel C Hagan, Laura S Porter

**Affiliations:** 1 Center for Health Promotion and Disease Prevention Edson College of Nursing and Health Innovation Arizona State University Phoenix, AZ United States; 2 Clinical Research Division Fred Hutchinson Cancer Research Center Seattle, WA United States; 3 Edson College of Nursing and Health Innovation Arizona State University Phoenix, AZ United States; 4 Counseling and Counseling Psychology College of Integrative Sciences and Arts Arizona State University Phoenix, AZ United States; 5 Psychiatry and Behavioral Sciences School of Medicine University of Washington Seattle, WA United States; 6 Division of Public Health Sciences Fred Hutchinson Cancer Research Center Seattle, WA United States; 7 Department of Psychology University of Washington Seattle, WA United States; 8 Department of Psychology Columbia University New York, NY United States; 9 Psychiatry and Behavioral Sciences Rush Medical College Rush University Chicago, IL United States; 10 Psychiatry and Behavioral Sciences School of Medicine Duke University Durham, NC United States

**Keywords:** ecological momentary assessment, smartphone, mobile phone, communication, disclosure, affect

## Abstract

**Background:**

Interpersonal communication is critical for a healthy romantic relationship. Emotional disclosure, coupled with perceived partner responsiveness, fosters closeness and adjustment (better mood and relationship satisfaction). On the contrary, holding back from disclosure is associated with increased distress and decreased relationship satisfaction. Prior studies assessing these constructs have been cross-sectional and have utilized global retrospective reports of communication. In addition, studies assessing holding back or perceived partner responsiveness have not taken advantage of smartphone ownership for data collection and have instead required website access or use of a study-provided device.

**Objective:**

This study aimed to examine the (1) usability and acceptability of a smartphone app designed to assess partner communication, closeness, mood, and relationship satisfaction over 14 days and (2) between-person versus within-person variability of key constructs to inform the utility of their capture via ecological momentary assessment using the participants’ own handheld devices.

**Methods:**

Adult community volunteers in a married or cohabiting partnered relationship received 2 smartphone prompts per day, one in the afternoon and one in the evening, for 14 days. In each prompt, participants were asked whether they had conversed with their partner either since awakening (afternoon prompt) or since the last assessment (evening prompt). If yes, a series of items assessed enacted communication, perceived partner communication, closeness, mood, and relationship satisfaction (evening only). Participants were interviewed by phone, 1 week after the end of the 14-day phase, to assess perceptions of the app. Content analysis was employed to identify key themes.

**Results:**

Participants (N=27; mean age 36, SD 12 years; 24/27, 89% female; 25/27, 93% white and 2/27, 7% Hispanic) responded to 79.2% (555/701) of the total prompts sent and completed 553 (78.9%) of those assessments. Of the responded prompts, 79.3% (440/555) were characterized by a report of having conversed with one’s partner. The app was seen as highly convenient (mean 4.15, SD 0.78, scale: 1-5) and easy to use (mean 4.39, SD 0.70, scale: 1-5). Qualitative analyses indicated that participants found the app generally easy to navigate, but the response window too short (45 min) and the random nature of receiving notifications vexing. With regard to the variability of the app-delivered items, intraclass correlation coefficients were generally <0.40, indicating that the majority of the variability in each measure was at the within-person level. Notable exceptions were enacted disclosure and relationship satisfaction.

**Conclusions:**

The findings of this study support the usability and acceptability of the app, with valuable user input to modify timing windows in future work. The findings also underscore the utility of an intensive repeated-measures approach, given the meaningful day-to-day variation (greater within-person vs between-person variability) in communication and mood.

## Introduction

Interpersonal communication is critical to the development and maintenance of romantic relationships [1]. The manner in which partners convey verbal and nonverbal information to each other plays a major role in the psychological functioning of both individuals and their relationship as a whole [2]. Much attention has been paid to disclosure, the act of revealing inner experiences to an interaction partner [3]. Disclosure is part of a larger interactional process that can lead to intimacy if it is met with responsive listening, due to perceptions of being cared for and understood [4]. Indeed, open discussions, the expression of thoughts and feelings, and responsiveness have all been found to be associated with increased intimacy and, in turn, increased relationship satisfaction and decreased psychological distress [5,6]. On the contrary, avoidance behaviors such as holding back have been found to be associated with lower intimacy, lower relationship satisfaction, and greater distress [5-13].

Much of the research informing the understanding of the links between communication and mood, as well as communication and relationship quality, has been based on cross-sectional survey studies in which communication is measured via a global retrospective report. Although informative, these measures are subject to recall biases and may be colored by a respondent’s current state [14]. Ecological momentary approaches afford assessment of experiences and behaviors in naturalistic contexts and in real time, which allow for the assessment of temporal processes [15-19]. Owing to the advances in technology, smartphone apps provide a platform to gather momentary data by assessing thoughts, feelings, and behaviors via notifications to respond to self-report surveys with language-based data [20]. Wheeler and Reis [21] referred to these assessments as “small events” and noted 3 different approaches to the timing of assessment: interval-contingent recording, signal-contingent recording, and event-contingent recording. The focus here is on the signal-contingent recording, a method by which subjects are prompted to report on a recent experience or event when a signal (ie, a smartphone notification) is received, in this case, on a random schedule within a fixed time interval. The advantages of the signal-contingent recording approach are that the report is close in time to the event, thus reducing recall errors and the likelihood of reappraisal. The disadvantages are that the notifications may be intrusive, and rarer events are less likely to be captured [21].

The primary purpose of this pilot project was to assess the usability and acceptability of a smartphone app designed to gather twice-daily reports of communication with a romantic partner as well as mood and closeness and daily reports of relationship satisfaction. The project was distinct from past research in multiple aspects. First, although the assessment of disclosure and responsiveness is not novel [22,23], to the authors’ knowledge, no study has assessed holding back from disclosure using a smartphone-based ecological momentary assessment (EMA). Second, a variety of communicative behaviors (eg, enacted and perceived disclosure, holding back, support provision) were captured with regard to the general conversation and not tied to specific concerns as is done in much of the medical literature, for example, in the work designed to capture partner responses to patient pain [24]. Third, most of the previous studies assessing partner communication using EMAs utilized paper-and-pencil diary methods, web-based methods wherein participants were instructed to log in and provide reports at certain times of the day, or electronic devices provided in the study [15,25,26]. In this study, we utilized the advantage of the ubiquity of smartphone ownership in the United States [27] to prompt responding on a device that participants are likely to have with them or close at hand. Thus, participants do not need to learn the mechanics of an unfamiliar device nor do they have to carry a separate device that could be bothersome or unwieldy. Project costs were also reduced.

To capture the constructs of interest, we used the LifeData platform, which is a template-based website that affords easy and economical creation of a smartphone app downloadable on iOS and Android platforms. In this study, the approach to the examination of usability and acceptability was both quantitative and qualitative. App-derived user data were used to examine the percentage of notifications that were responded to and completed. We also assessed the frequency of respondents reporting an interaction with their partner and whether this varied by the time of day. A qualitative analysis of semistructured interview questions (posed 1 week after completion of the 14-day EMA) identified participants’ perceptions of the ease of navigation and convenience of using the app.

Secondarily, we sought to examine the between-person vs within-person variability of key constructs (eg, disclosure, holding back, closeness, mood, and relationship satisfaction). We expected relationship satisfaction to differ between persons and be more stable over time within persons and hence only assessed that construct once per day, in the evening. Daily experience methods are ideal for the examination of within-person processes [15-17]. We assumed that mood would vary within persons based on past research [28,29]. The examination of variability of communication items was exploratory. Greater within-person variability relative to between-person variability would provide support for the utility of examining these variables repeatedly and in real time.

## Methods

### Participants

All procedures were approved by the Institutional Review Board (IRB) of Arizona State University. Participants were recruited from ResearchMatch, a free and secure registry that matches scientific studies to willing volunteers. Volunteers provide basic demographic and health information and agree to be contacted if they are a match for specific studies. At the time of writing this paper (April 14, 2020), ResearchMatch had 768 active studies, 8298 researchers across 169 institutions, and 146,987 volunteers.

Screening occurred in 2 stages. On the basis of the available demographic data, volunteers who were aged 18 years and above and residing in either North Carolina (NC) or Washington (WA) state were identified, mirroring the recruitment sites for a larger study to follow. The 1172 volunteers who met these criteria were sent an approach message conveying this study’s title and purpose, an overview of procedures, and the full inclusion criteria (18 years and above, residing in NC or WA, married or in a committed and cohabiting relationship of at least one year, ability to speak and understand English, and ownership of an iOS or Android smartphone).

Over a 2-week recruitment period, 149 of the 1172 matches responded to the approach message, with 104 conveying a willingness to be contacted and 45 declining further contact. Reasons for the decline were self-perceived ineligibility (n=29), lack of interest (n=8), lack of time (n=4), and no reason (n=4). The remaining 1023 matches did not respond to the approach message within the 2-week recruitment time frame and therefore were not pursued further. Of the willing 104 matches, 2 matches invited their spouse/partner to participate through IRB-approved snowball sampling. Although it was informative to know that these partners were willing to participate given the plans to recruit couples for a larger study, only 1 member of each of these 2 dyads was included in this analysis sample to ensure data independence.

Eligible volunteers responding affirmatively to the ResearchMatch contact message (and the two partner referrals) were contacted by phone to verify eligibility and confirm willingness to download the smartphone app: 2 declined participation, 7 were deemed ineligible via telephone screen, 13 did not have the correct contact information (unreachable), 54 did not respond to phone contact, and 30 were enrolled; 3 participants were excluded from the analyses: 2 as described earlier (one randomly from each of the 2 enrolled couples) and 1 who provided no data. This resulted in an analysis sample of 27 individuals.

### Procedures

Following consent, participants were instructed to download a free smartphone app called *RealLife Exp*, designed specifically for the study using LifeData, a web-based app development system. The project manager (second author) guided participants through the download process over phone. Upon download and registration, participants began receiving notifications to complete assessments twice daily for 14 days: once in the afternoon, between 1:00 PM and 2:00 PM, and once in the evening, between 7:30 PM and 8:30 PM (local time). Notifications were set to arrive randomly within these time windows. These time frames were chosen because we assumed that conversations with partners would be less likely to occur in the early morning. The evening time point was seen as not too late but sufficiently late to capture evening/dinner conversations. The time windows to begin each assessment were 45 min in length. Specific items and the constructs they were designed to assess are described in the following sections.

Participants could earn up to US $50 for completing all parts of the study: US $42 for completion of the smartphone-based assessments and US $8 for the follow-up phone interview. If they responded to 80% of the notifications or more, they received the full amount of US $42. If they responded to less than 80% of the notifications, they received US $1.50 per completed notification. The payment was in the form of an Amazon gift card sent via email.

#### Demographics

An initial assessment included questions to gather demographic characteristics such as age, sex, race, ethnicity, and length of the relationship with the partner.

#### Communication With Partner

At each assessment, participants were asked whether they had talked to their partner since waking up (afternoon assessment) or since the last set of questions (evening assessment). Those responding “yes” were asked a series of follow-up questions about the conversation to assess their own communicative behavior and perceptions of their partner’s communicative behavior. Disclosure and holding back were adapted from the Emotional Disclosure Scale [30]. Disclosure was assessed via a single item, “To what extent did you express your feelings during this conversation?” A parallel item assessed perceived partner disclosure, “To what extent did you feel that your partner expressed his/her feelings?” Holding back was also assessed with a single item, “To what extent did you hold back from expressing your feelings?” Additional items assessed facets of responsiveness: “To what extent did you support your partner?” “To what extent did you understand your partner?” “To what extent did you feel that your partner supported you?” and “To what extent did you feel that your partner understood you?” All of these items were rated on a 1 (*not at all*) to 5 (*a lot*) scale.

#### Closeness

Closeness was assessed with a single item, “How close do you feel to your partner right now?” Ratings were made on a 1 (*not at all*) to 5 (*extremely*) scale.

#### Mood

Mood was measured using an abbreviated version of the Profile of Mood States [31], following Cranford et al [28]. Three items formed each of the 4 subscales: anxious mood (anxious, on edge, and uneasy), depressed mood (sad, hopeless, and discouraged), anger (angry, resentful, and annoyed), and vigor (vigorous, cheerful, and lively). Ratings were made on a 1 (*not at all*) to 5 (*extremely*) scale, and the time referent was “right now.” For example, “How on edge do you feel right now?”

#### Relationship Satisfaction

**Figure 1:**
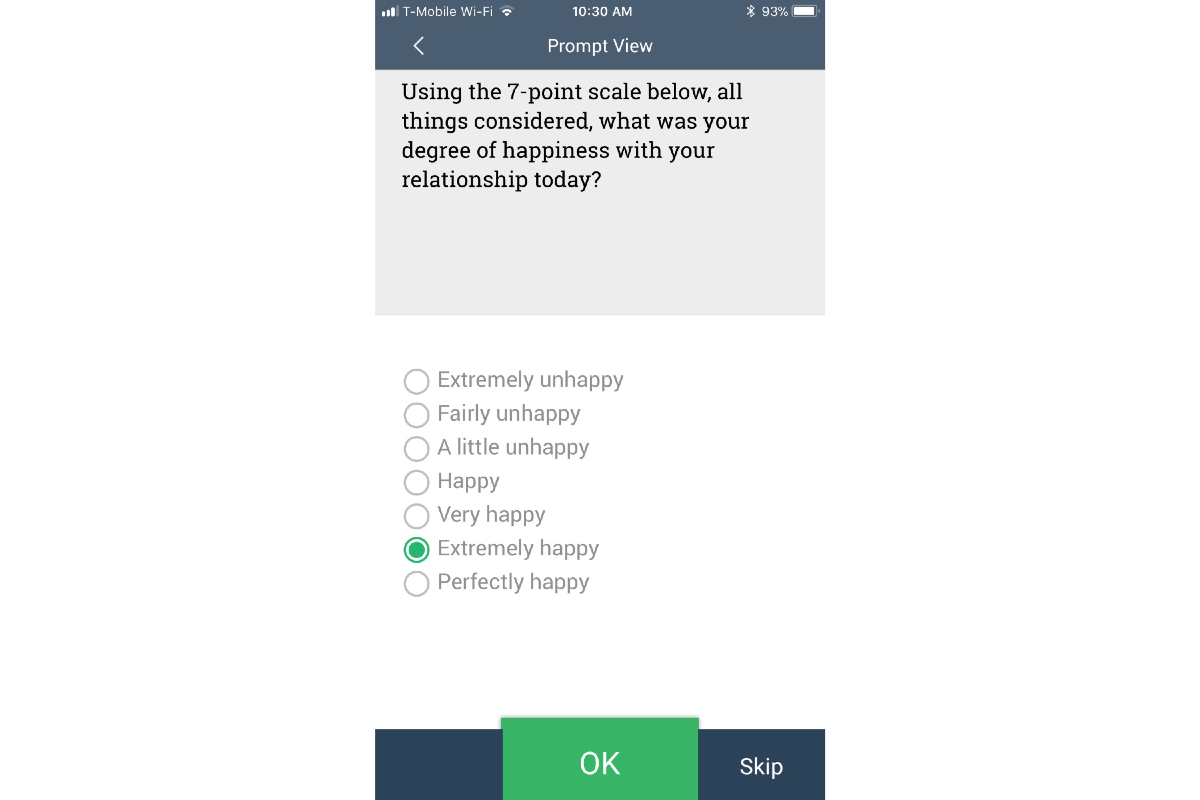
Screenshot of the app.

Relationship satisfaction was assessed with a single item from the Dyadic Adjustment Scale,[32], specifically item 31, following Auger et al [33]. This item was posed only at the evening assessment. Participants were asked, “All things considered, what was your degree of happiness with your relationship today?” Following the standard scale, options ranged from *extremely unhappy* to *perfectly happy*, coded from 1 to 7. See Figure 1 for a screenshot of this question.

### Follow-Up Interview

One week after the end of the 14-day EMA phase, participants were contacted by phone by the second author for a follow-up interview to assess perceptions of the app. Two questions were closed-ended, one to assess ease of use and the other to assess convenience, both indicators of acceptability. The other questions were open-ended, one to assess the convenience of notification timings (another indicator of acceptability) and the other to assess the ease of navigation (an indicator of usability or how well the app functions; Table 1). In posing the open-ended questions, the interviewer probed for clarification as necessary and took detailed notes, including verbatim speech.

**Table 1:** Measures of usability and acceptability.

Measures			Source		Type of data
**Usability (completion and navigation)**					
	Among notifications sent, the number responded to	App-derived user data		Objective, quantitative	
	Among notifications sent, the number completed	App-derived user data		Objective, quantitative	
	How easy was it to navigate within the app? (open-ended)	Follow-up interview		Qualitative	
**Acceptability (ease and convenience)**					
	On a scale of 1-5, how easy was it for you to use the app?	Follow-up interview		Subjective, quantitative	
	On a scale of 1-5, how convenient was it for you to use the app?	Follow-up interview		Subjective, quantitative	
	How convenient were the notification timings? (open-ended)	Follow-up interview		Qualitative	

### Analyses

#### Quantitative

Univariate descriptive statistics were used to summarize the sample’s demographic characteristics, EMA response and completion rates, and ratings of the app using SPSS 24.0. Descriptive statistics were also used to characterize the sample with respect to communication, closeness, mood, and relationship satisfaction items. To capture the proportion of the total variance in each item attributable to between-person differences vs within-person (ie, day-to-day) variability, we computed intraclass correlation coefficients (ICCs) from minimum norm quadratic unbiased estimation (MINQUE) estimates of variance components (between-person variance and within-person variance) using the minque package [34] in R. ICCs were computed for afternoon and evening assessments separately. Using Poisson regression models, the association of each background characteristic (measured at baseline) with the response rate (count of responses to prompts) and completion rate (count of completed assessments) was examined separately for afternoon and evening assessments. To test for afternoon vs evening differences in response and completion rates and in the frequency of speaking to one’s partner, we estimated single-predictor logistic regression models with bootstrap standard errors adjusted for a within-person clustering using the rms package [35] in R.

#### Qualitative Review

The second author conducted content analysis of the raw qualitative interview data, which included interviewer notes of responses to the open-ended items listed in Table 1 and direct participant quotations. The practical nature of the topic, relatively small sample size, and ease of capturing and interpreting participant responses to items and probes did not warrant an audio-recording or multiple coders. Methodological rigor was maintained by reviewing all of the detailed notes from each participant multiple times before generating preliminary codes, coding and categorizing identified issues by type and frequency of occurrence, identifying themes and refining codes in an iterative process, and continuing the analysis until no further themes were emerging from the data [36].

## Results

### Sample Characteristics

Table 2 displays the demographic characteristics of the analysis sample. The average age of participants was 36 (SD 12) years. Most participants identified as female (24/27, 89%), white (25/27, 93%), and non-Hispanic (25/27, 93%). The length of participants’ current marriage or partnered relationship varied greatly, with 22% (6/27) reporting relationships of 1 to 2 years and 15% (4/27) reporting being in their current relationships for 16 or more years.

**Table 2:** Demographic characteristics of the sample (N=27).

Variable^a^		Values
**Age (years)**		
	Mean (SD)	36.41 (11.99)
	Range	22-64
**Gender, n (%)**		
	Male	3 (11)
	Female	24 (89)
**Race, n (%)**		
	Black or African American	1 (4)
	White	25 (93)
	Multiracial	1 (4)
**Ethnicity, n (%)**		
	Hispanic or Latino/Latina	2 (7)
	Non-Hispanic	25 (93)
**Length of relationship in years, n (%)**		
	1-2	6 (22)
	3-5	4 (15)
	6-10	7 (26)
	11-15	6 (22)
	>16	4 (15)

^a^The length of the relationship was assessed categorically.

### App-Derived Usability Metrics

Table 3 presents app-derived usability metrics. Among 701 total notifications sent across participants and both afternoon and evening assessments, 555 (79.2%) were responded to and 553 (78.9%) were completed. These values did not differ as a function of the assessment time point (*P*>.27). In addition, counts of responses to afternoon and evening EMA prompts and counts of afternoon and evening EMA completion rates were unrelated to age, gender, race (dichotomized as white vs black or multiracial), ethnicity, or relationship length (*P*>.64).

Among the prompts responded to, 79.3% (440/555) were characterized by a report of having conversed with one’s partner (either since waking up for the afternoon prompt or since the last assessment for the evening prompt). This rate was higher for the evening vs afternoon time point (86% vs 72%); Wald *z* from Poisson regression was 2.79 (*P*=.005).

**Table 3:** App-derived usability metrics.

Usability metrics	Total	Afternoon	Evening	*P* value
Number of notifications sent, n	701	345	356	N/A^a^
Notifications responded to, n (%)	555 (79.2)	268 (77.7)	287 (80.6)	.28
Assessments completed, n (%)	553 (78.9)	268 (77.7)	285 (80.1)	.37
Conversed with partner since waking up or last notification (prompts responded to), n (%)	440 (79.3)	193 (72.0)	247 (86.1)	.005

^a^N/A: not applicable.

### Self-Report Ratings of Acceptability

Of the 27 participants, 26 completed the follow-up interview. Mean ratings of ease of use and convenience of the app fell well above the midpoint of the 1-5 (*not at all* to *extremely*) scale: mean 4.39 (SD 0.70) for “How easy was it for you to use the app?” and mean 4.15 (SD 0.78) for “How convenient was it for you to use the app?”

### Content Analysis of Responses to the Open-Ended Interview Questions

In what follows, we describe themes derived from content analysis of the open-ended interview items listed in Table 1. Representative participant quotations are included to illustrate salient findings. Two broad categories emerged: (1) technical functioning and navigation and (2) response convenience, notification timings, and session active window.

### Technical Functioning and Navigation

Overall, participants found the app to be very streamlined and user-friendly, perceiving it as a useful tool that functioned without technical difficulty. Notifications arrived as planned and were visible on the home screen of the phone and as an alert on the app icon. Navigation was reported to be simple for the most part, with an intuitive process to move forward:

App was very simple...could not be made easier.

Just hit OK and move forward, really easy.

Very basic and simple, nice to have it on the home screen.

Specific actions within the navigation process elicited comments. Some found the skip option useful, particularly if the answer was unknown or if a respondent felt uncomfortable sharing the information in question. The “go back” function provoked some frustration. Participants who wanted to review their previous answer but then decided not to change it after all had to reselect the same answer to move forward:

If I went back, I had to re-click my answer even if I didn’t want to change it.

There were several indications that the download process (which the project manager instructed the participant to do step-by-step over the telephone) was quite complicated and time-consuming. There was a notable difference between the perceived complexity of the download and the reported simplicity of using the app, suggesting that direct guidance and a user brochure could greatly facilitate this process:

Very easy...the setup that you walked me through, that was harder … then it all worked fine.

### Response Convenience, Notification Timings, and Session Active Window

In general, participants indicated that delivery of the assessment via the app was very convenient, insofar as they typically had their smartphone available and usually saw or heard the notification or looked for it within the expected timeframe.

Participants reported it to be much easier if they responded from the phone’s home screen, rather than going into the app itself and seeing the alert there and then beginning the survey (if the notification was missed):

I liked that you could just swipe the notification and go right into it, much easier than if you missed it and the notification went away, then had to go to the app and see the alert.

Participants also said that being active in another app could impede response to the notification because some apps do not allow new notifications while open.

Perceptions regarding session timings were largely driven by differences in personal schedules. There was a marked preference for the evening session as most respondents were more able to interrupt their activities at that time. One primary recommendation was to change the timing of the afternoon session:

It was really hard for me in the afternoon. I would rather it was coming either mid-morning or at a more traditional lunchtime.

I tended to remember the evening one more, so I would check the phone more periodically.

The 45-min window during which the notification remained active (it expired and was no longer accessible after 45 min) was too short for most of the participants. The primary recommendation was to increase the session active window to at least one hour and preferably to 1.5 hours to accommodate events that last an hour:

Biggest issue, expired too quickly!

The window was way too short and many times I found it difficult to answer in the time.

Randomization of the session timings within an hour window was frustrating to many participants. Some reported resorting to setting alarms and “waiting” for the notification to arrive. Irritation with randomization tended to increase over the 14-day activity. The primary issue was not knowing when the notification would arrive and anxiety over “missing” it:

It was a little vexing...I set an alarm so I would be ready, but since it always changed the time it was really a little crazy.

### Descriptive Statistics for Key Variables

Table 4 displays means, standard deviations, and ICCs for key constructs, as a function of the time point (afternoon or evening). Levels of enacted and perceived disclosure were relatively high, as were levels of support provision, understanding, and perceived partner support and understanding. Levels of holding back were low. To reiterate, all of these items were rated on a 1-5 (not at all to a lot) scale. Relationship satisfaction, rated on a 1-7 (extremely unhappy to perfectly happy) scale, was moderately high on average (mean 5.20, SD 1.28). Levels of vigor were below the scale midpoint of 3 on average. Levels of anxiety, anger, and depressed affect were below a score of 2 on average.

The ratio of between-person variance to the total variance (where total variance=between-person variance + within-person variance) is reflected by the ICC values listed in the fifth column of Table 4. As shown in Table 4, ICCs for EMA measures were generally <0.40, indicating that the majority of the variability in each measure was at the within-person level, rather than at the between-person level, suggesting that there was meaningful day-to-day variation in these variables. Notable exceptions were enacted disclosure (ICCs=0.46 and 0.42 for afternoon and evening assessments, respectively), closeness (ICCs=0.41 and 0.40, respectively), and relationship satisfaction (ICC=0.59), indicating that between-person differences in these variables were relatively more stable across the 14-day period.

**Table 4:** Means, standard deviations, and intraclass correlation coefficients for smartphone-assessed constructs.

Variable and time of day^a^		N	Value, mean (SD)	ICC^b^
**To what extent did you express your feelings?**				
	Afternoon	193	3.67 (1.22)	0.46
	Evening	245	3.64 (1.17)	0.42
**To what extent did you feel that your partner expressed his/her feelings?**				
	Afternoon	189	3.74 (1.16)	0.18
	Evening	242	3.87 (1.08)	0.24
**To what extent did you hold back from expressing your feelings?**				
	Afternoon	193	1.62 (1.04)	0.23
	Evening	246	1.77 (1.06)	0.37
**To what extent did you support your partner?**				
	Afternoon	189	3.78 (1.25)	0.33
	Evening	243	3.91 (1.09)	0.38
**To what extent did you understand your partner?**				
	Afternoon	189	3.90 (1.09)	0.25
	Evening	241	3.91 (1.02)	0.29
**To what extent did you feel that your partner supported you?**				
	Afternoon	190	3.85 (1.22)	0.19
	Evening	242	3.81 (1.08)	0.31
**To what extent did you feel that your partner understood you?**				
	Afternoon	190	3.70 (1.20)	0.14
	Evening	241	3.76 (1.06)	0.17
**How close do you feel to your partner right now?**				
	Afternoon	267	4.04 (0.93)	0.41
	Evening	283	4.04 (0.91)	0.40
**POMS^c^ vigor subscale**				
	Afternoon	268	2.82 (0.85)	0.22
	Evening	284	2.59 (0.81)	0.25
**POMS anxiety subscale**				
	Afternoon	268	1.76 (0.90)	0.30
	Evening	284	1.66 (0.81)	0.32
**POMS anger subscale**				
	Afternoon	268	1.48 (0.81)	0.27
	Evening	284	1.50 (0.75)	0.26
**POMS depressed affect subscale**				
	Afternoon	268	1.60 (0.84)	0.32
	Evening	284	1.52 (0.74)	0.33
**Relationship satisfaction**				
	Evening	285	5.20 (1.28)	0.59

^a^All items were rated on a 1-5 scale except for relationship satisfaction which was rated on a 1-7 scale.

^b^ICC: intraclass correlation coefficient.

^c^POMS: Profile of Mood States.

## Discussion

### Principal Findings

The primary goal of this pilot project was to examine the usability and acceptability of a smartphone app designed to assess communication with a romantic partner, closeness, mood, and relationship satisfaction repeatedly over the course of 14 days. The app was rated as easy to navigate, and the response rate was quite good. Of the 701 total notifications sent, 555 (79.2%) were responded to and 553 (78.9%) were completed. Comparing these rates with those reported in the literature is challenging, given the wide variability in the frequency of prompts, number and content of items posed, and sample characteristics. Incentive structures also likely vary. However, in general, the completion rates fell within the ranges reported by other research teams [37], in some cases higher by 8% to 14% [38-40] and in other cases lower by 4% to 7% [41]. These differences may, in part, be explained by differences in numbers of items, for example, the battery was somewhat longer than that described by Perndorfer et al [41].

With regard to acceptability, the app was rated as convenient to use on average (mean 4.15 on a 1-5 scale). However, qualitative analyses provided a more nuanced understanding of the perceived acceptability of the app. Participants expressed difficulty with 3 aspects related to timing: (1) The afternoon prompt came between 1:00 PM and 2:00 PM, which may have been difficult for employed participants due to work-related demands. (2) Notification times (signals to respond) were randomized within a 1-hour period. Inability to anticipate the notification’s arrival was frustrating. (3) The active time window to begin each assessment was 45 min, which participants felt was too short. On the basis of this feedback, modifications were made to the app for a larger ongoing study of couples coping with cancer. In the ongoing study, notifications are delivered at fixed times (at noon and 8:00 PM), and the time window to complete assessments is 2 hours. We also added reminders (a LifeData feature not available at the time of the pilot) that arrive every 20 min within the open window. Further modifications were made in response to the technical navigation issues raised. For example, the app user guide was refined to clearly describe how and when to use the skip and go back functions.

One possible drawback of signal-contingent recording is that infrequent behaviors might not be captured [21]. Indeed, we did not know before conducting this study whether conversations with a spouse or partner would occur during the periods in question. Findings suggest that partner conversations are sufficiently frequent to warrant an assessment of the occurrence and nature of those conversations using EMA methods. Among the prompts responded to, 79% were characterized by a report of having conversed with one’s partner (either since waking up for the afternoon prompt or since the last assessment for the evening prompt). This value was significantly higher for evening vs afternoon reports. Most participants were likely away from home at work during the day time, or if at home, may have been engaged in activities apart from their partner, making daytime conversations less likely. However, this is a conjecture, as we did not formally assess the employment status (though it was mentioned by some participants in the interview).

When conversations did occur, they were characterized, on average, by moderately high levels of disclosure, both enacted and perceived. Participants also saw themselves, in general, as being supportive and understanding, and in turn as receiving support and understanding. Holding back was less likely to occur. While exact comparisons to other reports in the literature are difficult to draw given inconsistencies in the communicative behaviors measured and, in some cases, the use of different rating scales, the relative frequency of the behaviors is generally in line with reports derived from traditional questionnaire measures of communication. For example, Porter et al [42] observed moderately high levels of disclosure and low levels of holding back among patients with gastrointestinal cancer and their spouses.

A secondary goal of this study was to determine between-person variability vs within-person variability of the study variables. ICCs underscore the utility of the EMA approach for the measurement of the communication items, all indicating greater within-person variability than between-person variability. Disclosure showed lower within-person variability than other communication measures, perhaps reflecting a dispositional tendency to express emotion across time and situations. Similar to the majority of communication behaviors, measures of anger, anxiety, depressed mood, and vigor showed considerable day-to-day variability within persons. Closeness and relationship satisfaction showed greater stability over the 14-day period. The relationship satisfaction finding is consistent with that reported by Gadassi et al [26] who administered the same single item. These findings are also in line with this study’s expectation that this construct would vary less over time within persons than between persons.

### Limitations

Limitations of this pilot study must be considered. By design, the number of participants was small. The sample was comprised largely of non-Hispanic white women, limiting the generalizability of the results. As women tend to be more emotionally expressive than men, [43], this could explain the fairly high levels of enacted disclosure and low levels of holding back. The recruitment source, ResearchMatch, also limits generalizability. This website matches researchers to willing volunteers, that is, persons open to the idea of research and perhaps motivated to earn incentives for participation. A published analysis of the ResearchMatch volunteer database (N=15,871) indicated that 81% of volunteers identified as white and 95% as non-Hispanic [44]. The average age was 38 years, and most volunteers (73%) were female. The demographic composition of this small sample mirrors this larger pool.

It is also important to note that this study, while focused on partner communication, was not dyadic in nature. This approach was chosen to hasten recruitment and based on the assumption that usability and acceptability data from one member of a dyad would be sufficient to inform the next steps for a larger study with dyads. Relatedly, partner characteristics were not assessed nor were participants asked to report on partner characteristics including demographic characteristics. Therefore, it is not known whether members of each couple were of the same or different sex/gender. We also relied entirely on participant self-reports of their own behavior and reports of their partner’s behavior. Thus, it is not possible to examine concordance between self- and partner reports of communicative behavior.

As described in the Introduction, much of the research on couple communication has been designed to assess communication in reference to a specific concern or topic. This is often the case in laboratory-based studies wherein couples are asked to discuss either a relevant shared stressor or a conflictual topic. It has also been the case in numerous questionnaire-based assessments of holding back in which couples are asked to rate the extent to which they (1) disclosed and (2) held back from disclosing a number of different illness-related concerns [45-49]. In this study, the participants were not recruited based on a common stressor or illness. Conversations were not constrained to a specific topic nor were the participants asked to report on the topic. Therefore, we do not know what was discussed nor do we have a sense of the valence of each conversation. These contextual variables may be important to examine as moderators in future research. Perceived lack of responsiveness from one’s partner, for example, may be more deleterious in the context of a highly stressful topic, such as serious illness or relationship distress, vs in the context of daily hassles.

Despite the study’s limitations, findings from this pilot project lend support for the use of smartphone apps to assess communication in real time and in naturalistic settings. They also underscore the advantages of using a web-based template for app creation, a highly affordable option as opposed to hiring a programmer or developer. On the basis of the usability data and feedback from participants, this smartphone app has been since adapted for use with a larger sample of patients with cancer and their cohabiting partners/spouses. The larger study is still in process but initial results suggest strong completion rates and acceptability of the app. Future interventions designed to train couples in adaptive communication could potentially make use of EMA data such as these to inform targeted approaches and to monitor the response to the intervention.

## Abbreviations

EMA: ecological momentary assessment
ICC: intraclass correlation coefficient
IRB: Institutional Review Board
